# The effectiveness of Interpersonal Psychotherapy-Adolescent Skills Training for adolescents with depression: a systematic review and meta-analysis

**DOI:** 10.3389/fpsyt.2023.1147864

**Published:** 2023-07-31

**Authors:** Kewei Zheng, Huimin Xu, Chunhui Qu, Xianghong Sun, Na Xu, Ping Sun

**Affiliations:** ^1^School of Humanities and Social Sciences, Binzhou Medical University, Yantai, China; ^2^Qingdao Mental Health Center, Qingdao, China

**Keywords:** Interpersonal Psychotherapy-Adolescent Skills Training, depression, adolescents, systematic review, meta-analysis

## Abstract

**Background:**

Interpersonal Psychotherapy-Adolescent Skills Training (IPT-AST) is a standardized depression prevention program for adolescents conducted in campus settings. The purpose of this review is to examine the randomized controlled trials of IPT-AST for the prevention of adolescent depression in the past 20 years.

**Methods:**

A systematic search of relevant electronic databases (PubMed, WOS, Embase, PsycINFO, the Cochrane Library, CNKI and WANFANG DATA) and study reference lists was conducted. Any study investigating the effectiveness of IPT-AST in 12- to 20-year-olds with depressive symptoms was eligible. Synthesis was via narrative summary and meta-analysis.

**Results:**

A total of 6 studies met the inclusion criteria. Meta-analysis results showed a remarkable improvement in patients’ depressive symptoms after IPT-AST intervention (WMD = −5.05, 95% CI = −8.11 to −1.98, *p* < 0.05, *I*^2^ = 77%). Six month follow-up data showed that the intervention outcomes of IPT-AST remained significant (WMD = −3.09, 95% CI: −5.23 to −0.94, *p* < 0.05, *I*^2^ = 57%).

**Conclusion:**

This meta-analysis showed that IPT-AST was effective in adolescents with depressive symptoms at post-prevention and at 6-month follow-up. However, these conclusions are cautious, as they are based on a small number of studies and the presence of author duplication. Future studies should use multi-center, large-sample randomized controlled trials to evaluate the efficacy of IPT-AST for preventing depression in adolescents.

**Systematic review registration:**

https://www.crd.york.ac.uk/prospero/, identifier CRD42023393047.

## 1. Introduction

Adolescent depression is a very common mental illness that can seriously damage the physical and mental health of adolescents (1). Epidemiological surveys show that the prevalence of depression in adolescents ranges from 0.4 to 8.3%, and the prevalence of depressive symptoms ranges from 22 to 60% (2, 3). The ages of 12 to 17 are the high-risk period for adolescent depression (4). A research from Lancet also indicates that approximately one in every five adolescents experiences a depressive episode before the end of puberty (5). Although medication and psychological therapy are commonly used to treat depression and they have been proven to be effective treatments for depression (6–11), evidence suggests that these treatment methods have been found to work for only about 50–60% (12). Therefore, it is necessary to reduce the incidence of depression in adolescents and to prevent it.

The Institute of Medicine (IOM) defines prevention as preventive interventions taken when symptoms have not yet met diagnostic criteria but are present (13). A randomized controlled trial in 2021 showed that timely prevention was effective in reducing depressive symptoms in adolescents (14). Currently, prevention like psychotherapy and psychoeducation are widely employed (13). There is evidence that psychotherapy can effectively prevent depression by alleviating depressive symptoms (15). For adolescents, many studies have shown that poor interpersonal relationships are one of the significant factors contributing to the development of depression in adolescents (16–18). Several studies support the idea that improving relationships may be an important breakthrough in alleviating depressive symptoms and preventing depression (19–21). Interpersonal Psychotherapy - Adolescents (IPT-A) is precisely an interpersonal relationship-based prevention and treatment approach for adolescent depression (22, 23) and focuses on four interpersonal concerns (interpersonal conflicts, role transitions, grieving reactions, and lack of interpersonal relationships) in order to reduce depressive symptoms and enhance interpersonal abilities (24). A meta-analysis showed that IPT-A has a positive effect on alleviating depressive symptoms and preventing depression in adolescents (25). However, traditional psychotherapy such as IPT-A often requires a professional environment, which makes it difficult for some adolescents to obtain timely and effective interventions due to reasons such as distance, cost, or stigma (26).

Interpersonal Psychotherapy-Adolescent Skills Training (IPT-AST) is a school-based preventative approach to manage depression in adolescents (27). It is based on IPT-A and emphasizes that schools are an ideal environment for teaching social skills to adolescents and preventing depression (28). It advocates for improving adolescents’ understanding of depressive symptoms and interpersonal relationship problems through group therapy and psychological education (27). IPT-AST not only overcomes the limitations of traditional psychotherapy locations, but also group therapy delivered in school helps alleviate the adverse effects of cost and shame on adolescents (28). A meta-analysis showed that implementing depression prevention programs in school environments helps adolescents alleviate depressive symptoms and achieve prevention goals (29). In order to test the effectiveness of IPT-AST in preventing depression, Annette (30) implemented it on campus in 14 adolescents aged between 14 and 18 years, who had depressive symptoms but did not meet the diagnostic criteria yet, and the results showed that IPT-AST could effectively prevent depression in adolescents, reduce depressive symptoms and obtain more social support from friends. Therefore, implementing IPT-AST in school for adolescents who have just developed depressive symptoms seems to be an effective measure for preventing depression.

Although IPT-AST has been shown to be effective, the duration of its effect is unclear. Researchers have formed two different views based on multiple studies. Some studies suggest that the prevention effect can last for only 6 months. For example, a randomized controlled trial showed a significant preventive effect of IPT-AST, but its effect did not continue beyond 6 months (31). However, other studies have shown that the preventive effect of IPT-AST can last for 6 months or even longer. Young compared the prevention effects of IPT-AST and School Counseling. The results showed that compared with School Counseling, IPT-AST significantly alleviated the depressive symptoms of adolescents, and the effects lasted for 6 months or longer (32). Currently, there is no systematic review of the duration of prevention effect of IPT-AST. This meta-analysis will comprehensively examine the preventive and follow-up effects of IPT-AST on adolescent depression by including as many randomized controlled trials as possible.

## 2. Methods

### 2.1. Protocol and registration

The primary design of present review was registered on PROSPERO (CRD42023393047), and was conformed to the Preferred Reporting Items for Systematic Reviews and Meta Analyses (PRISMA) guidelines regarding evidence selection, quality assessment, evidence synthesis, and research reporting [Hutton et al. (33)].

### 2.2. Search strategy

We use keywords searching in multiple electronic database from January 1,2000 to October 30,2022. The keywords we used for searching include Interpersonal Psychotherapy-Adolescent Skills Training or IPT-AST; and Depression or Depressive symptoms; and Adolescen* or Teen* or Youth* or Young people and we also screened reference lists for additional articles. The databases include PsycINFO; Web of Science; EMBASE; Pubmed; the Cochrane Library; CNKI and WANFANG DATA.

### 2.3. Inclusion criteria

Studies that met the following criteria were included in the selection:

(1) Participants were adolescents (age from 12 to 20 years old),
(2) Depressive symptoms (scoring above cut-off on a standardized depression rating scale according to the original authors’ definition but does not meet diagnosis of depression),
(3) IPT-AST or minor modifications of IPT-AST,
(4) Compared to any control conditions,
(5) Randomized controlled trials (RCTs),
(6) When data were duplicated in different studies, newly published and data-complete studies were selected.

### 2.4. Outcomes

Because the IPT-AST is a group depression prevention program conducted on campus, we chose the Center for Epidemiologic Studies-Depression Scale (CES-D), a widely used self-report depression measure with strong psychometric properties with adolescents, to evaluate depression symptoms (34). In the included studies, the main indicator of efficacy was the difference in scores on the standardized scale after prevention and at follow-up.

### 2.5. Study selection

The search results are extracted and stored in the data management software. Two reviewers (KWZ and HMX) independently reviewed the identified studies and derived data from the enrolled studies. If there is disagreement, it is resolved through discussion and, if necessary, a third author is consulted to reach a final decision.

### 2.6. Data extraction

The outcomes of interest for this review were depression from validated scales. Two reviewers (KWZ and HMX) independently extracted the data. Relevant data for each measure was entered into Stata 14.0. We examined baseline and post-prevention scores between groups to assess the effectiveness of depression and follow-up effects. If sufficient data were not available in the article, the authors were consulted and additional information requested.

### 2.7. Assessment of study quality

Two reviewers (KWZ and HMX) independently evaluated the risk of bias in the enrolled studies using the Cochrane Collaboration’s Risk of Bias tool (33). The following criteria were assessed: sequence generation, allocation concealment, blinding of participants and care providers, blinding of outcome assessors, incomplete outcome data, selective outcome reporting and other sources of bias. Each criterion was evaluated as either “high,” “unclear” or “low” risk of bias according to the Cochrane Handbook (35).

### 2.8. Data items

The following study data were drawn: study characteristics (author, year, study type, follow-up); population characteristics (sample size, age, gender); experimental conditions (prevention group, control group); and outcomes (mean and standard error of scale scores at baseline, post-prevention, and follow-up).

### 2.9. Statistical analysis

The extracted data was stored in Stata 14.0. Checked for publication bias by Egger’s test. The Q test and I^2^ statistic were used to examine the heterogeneity, with *Q* > 0.05 and *I*^2^ ≤ 50% indicating low heterogeneity. Sensitivity analyses were performed by removing each eligible study to see whether the significance and the pooled effect size would be affected.

## 3. Results

### 3.1. Selection and inclusion of studies

**FIGURE 1:**
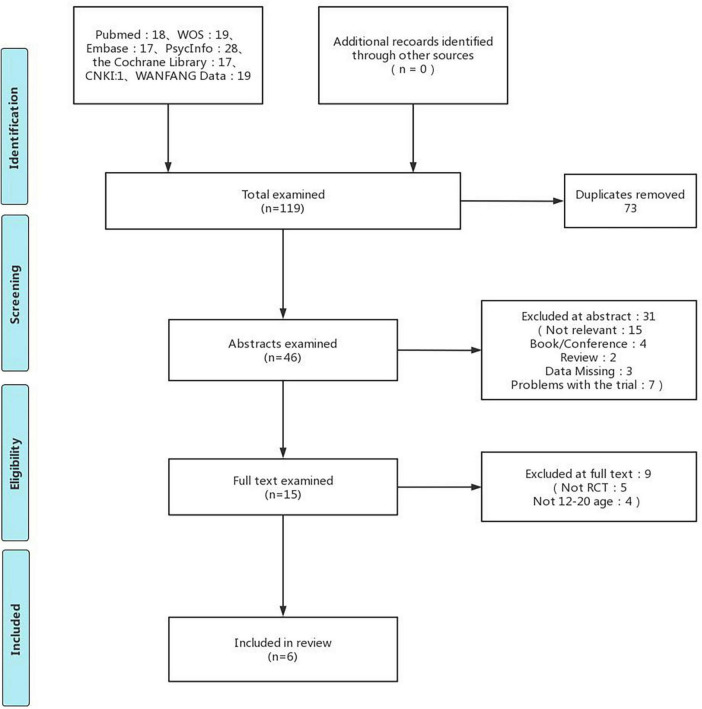
Flow diagram for evidence source search. Adapted with permission from (47), licensed under CC BY-NC 2.0.

After searching, a total of 119 studies were retrieved; after removing duplicates, 46 studies remained. Two reviewers (KWZ and HMX) performed the initial screening of titles and abstracts, and 31 were excluded, leaving 15 for full-text screening. Finally, 6 studies were included (Figure 1). 757 participants in total for all trials, with participants aged 11 to 18 years and predominantly female, including 366 in the experimental group and 391 in the control group.

### 3.2. Study characteristics

Among the six enrolled studies (28, 31, 36–39), the age range of the participants was 11–16 years with a mean age of 13.77 years (see Table 1). All studies were conducted in schools, with the experimental group using IPT-AST, five studies (28, 36–39) using school counseling or group counseling on campus as a control group, and one study (31) had a control group without any prevention approach. The studies all used the CES-D to measure participants’ depressive symptoms at baseline, post-prevention, and follow-up. For follow-up, all studies collected data from participants at 6 months of follow-up, and 2 studies (36, 39) collected results at 12 months and beyond.

**TABLE 1:** Summary description of studies reviewed.

References	Participants	Age mean	Prevention	Control	Outcome measures	Design	Follow-up length	Country, setting	Post severity of CES-D mean (SD)
Young et al. (35)	*n* = 174	14.01 (1.22)	IPT-AST involves 2 initial individual sessions and 8 weekly 90-min group sessions	GC	CES-D	RCT	6 months	America middle and high schools	8.97 (7.79)
Young et al. (47)	*n* = 174	14.01 (1.22)	IPT-AST involves 2 initial individual sessions and 8 weekly 90-min group sessions	GC	CES-D	RCT	6 months	America middle and high schools	9.68 (7.82)
Young et al. (36)	*n* = 41	13.4 (1.2)	IPT-AST involves 2 initial individual sessions and 8 weekly 90-min group sessions	SC	CES-D	RCT	6 months	America	9.75 (7.04)
Young et al. (37)	*n* = 41	13.37 (1.19)	IPT-AST involves 2 initial individual sessions and 8 weekly 90-min group sessions	SC	CES-D	RCT	6 months	America	6.30 (5.36)
Horowitz et al. (31)	*n* = 268	14.43 (0.7)	IPT-AST involves 2 initial individual sessions and 8 weekly 90-min group sessions	Control	CES-D	RCT	6 months	America suburban/rural high schools	17.42 (11.55)
Young et al. (28)	*n* = 41	13.4 (1.2)	IPT-AST involves 2 initial individual sessions and 8 weekly 90-min group sessions	SC	CES-D	RCT	6 months	America Catholic schools	6.3 (5.4)

IPT-AST, Interpersonal Psychotherapy-Adolescent Skills Training; GC, group counseling; SC, school counseling; CES-D, the self-report Center for Epidemiologic Studies–Depression Scale.

### 3.3. Risk of bias

**FIGURE 2:**
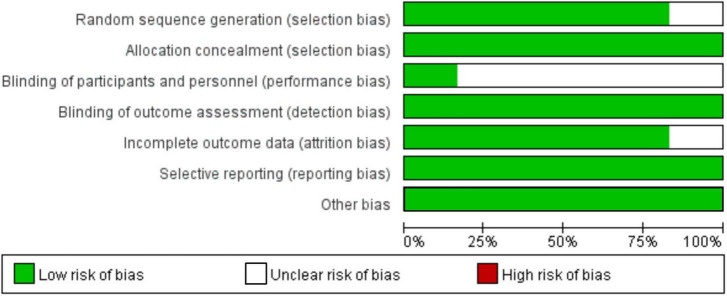
Risk-of-bias chart for studies included in the quantitative analysis.

The quality of included studies was assessed using the Cochrane Risk of Bias tool (40), and their results are presented (Figure 2). Five studies (28, 31, 36, 37, 39) used a random number table method for random grouping, and one study (38) did not describe the method of random assignment. Six researches explained allocation concealment, and subjects and researchers were unable to predict the outcome of allocation. Five studies (28, 36–39) did not describe in detail the method of blinding of investigators and subjects, and there may be bias. Six studies described methods for blinding study outcome evaluators. Of the follow-up results, five studies (28, 36–39) reported on the presence of missed visits/withdrawals and the reasons for missed visits/withdrawals.

### 3.4. Prevention effect of IPT-AST on adolescents with depression

#### 3.4.1. Depression (post-prevention)

**FIGURE 3:**
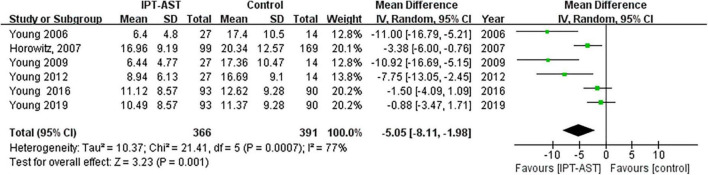
Forest plot showing the outcome of depressive symptoms after prevention.

For changes in depressive symptoms after the prevention, the results of all six studies were pooled for meta-analysis. The results showed significant improvement in depressive symptoms after the IPT-AST prevention (WMD = −5.05, 95% CI = −8.11 to −1.98, *p* < 0.05). See forest plot in Figure 3.

The results of the meta-analysis of the six trials had a high heterogeneity (*I*^2^ = 77%), which may be due, among other things, to the small number of included literature.

#### 3.4.2. Depression (follow-up)

**FIGURE 4:**
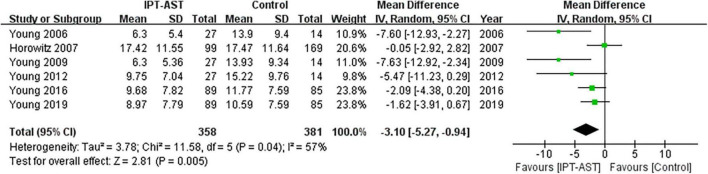
Forest plot showing the outcome of depressive symptoms at follow-up.

Six trials (*n* = 729) reported depression at 6-month follow-up; however, 18 individuals were lost to follow-up from prevention. The results showed a significant follow-up effect of IPT-AST for depressive symptoms (WMD = −3.09, 95% CI: −5.23 to −0.94; *p* < 0.05) and low heterogeneity between studies (*I*^2^ = 57%). See forest plot in Figure 4.

## 4. Discussion

This is the first meta-analysis evaluating the preventive and follow-up effects of IPT-AST on adolescent depression. By summarizing six studies, we found that IPT-AST as a preventative approach was effective in adolescents with depressive symptoms at post-prevention and at a 6-month follow-up. IPT-AST delivered in school overcomes the limitations of traditional treatment settings making it more accessible and promoted (27, 28). Adolescents are more familiar with and accepting of the school environment, so the prevention is easier to carry out there than in hospitals, treatment rooms, and other settings. In addition, IPT-AST focuses on interpersonal relationships which may be a key reason for its effectiveness in preventing depression in adolescents (5). IPT-AST encourages adolescents to practice social skills on campus, which allows them to cope with the interpersonal problems in daily life and maintain the preventive effects. These findings will provide school mental health educators and psychotherapists with new ideas for preventing depression in adolescents.

However, there are several limitations to this study which require caution in interpreting the results. Firstly, IPT-AST was exclusively evaluated in a limited number of schools within the United States; therefore, it is necessary to verify its efficacy in managing adolescent depression symptoms across diverse settings (beyond merely school-based environments) and cultural contexts in future research. Second, five of the six studies included in the meta-analysis belonged to the same research team, but these studies were not conducted at the same time and did not use the same participants and its data; therefore, these studies can be considered as different studies included in the meta-analysis. It is possible that the fact that IPT-AST is in an emerging phase has led to this situation, but this situation may also potentially limit the generalizability of our study findings. Therefore, it should be further explored in future studies. Third, the included studies did not examine the factors (age, country, ethnicity, etc.) that may influence the effectiveness of IPT-AST prevention, so these factors could be further explored in future studies.

Undoubtedly, the current research has systematically evaluated the preventive and follow-up effects of Interpersonal Psychotherapy-Adolescent Skills Training (IPT-AST), and has affirmed its effectiveness within a certain scope. With the outbreak of COVID-19, the concern for physical health has reached a new level along with the concern for mental health. Mental health concerns and research on adolescent populations have increased subsequently (41). One study (42) compared the psychological changes of adolescents before and after the epidemic and noticed that negative emotions among adolescents had significantly increased after the outbreak of the epidemic. Recent studies have also found that the incidence of depression among adolescents has increased during the spread of COVID-19 (43, 44). Therefore, it is crucial to timely prevent adolescent depression and reduce its incidence during this period. IPT-AST can meet this social demand, because it is a group-based depression prevention approach in a campus setting that greatly overcomes the limitations of traditional treatments and increases the efficiency and prevalence of depression prevention (13, 27, 45, 46).

## 5. Conclusion

This meta-analysis showed that IPT-AST was effective in adolescents with depressive symptoms at post-prevention and at 6-month follow-up. However, these conclusions are cautious, as they are based on a small number of studies and the presence of author duplication. Future studies should use multi-center, large-sample randomized controlled trials to evaluate the efficacy of IPT-AST for preventing depression in adolescents.

## Data availability statement

The original contributions presented in this study are included in this article/supplementary material, further inquiries can be directed to the corresponding authors.

## Author contributions

KZ and HX conceived the study design, searched and screened the article titles and abstracts, confirmed the data and statistical analyses, and responsible for the supervision and quality control. CQ, XS, PS, and NX drafted and supplemented the study methods. PS and NX revised their English and provided the assistance with data analysis. All authors provided the information on the direction of the study and the content of the manuscript and approved the final version of the manuscript.
